# Case Report: Intercostal Lymph Node Metastasis: A Case Report and Review of the Literature

**DOI:** 10.3389/fonc.2021.638948

**Published:** 2021-03-04

**Authors:** Yurong Zhou, Jinxuan Hou, Ning Meng, Staiculescu Daniel, Jiang Chen, Liying Xu

**Affiliations:** ^1^Department of Radiology, Zhongnan Hospital of Wuhan University, Wuhan, China; ^2^Department of Thyroid and Breast Surgery, Zhongnan Hospital of Wuhan University, Wuhan, China; ^3^Department of Thyroid and Breast Surgery, The Affiliated Hospital of Hangzhou Normal University Hangzhou, Hangzhou, China; ^4^Department of Radiation Oncology, Massachusetts General Hospital and Harvard Medical School, Boston, MA, United States; ^5^Department of General Surgery, Sir Run Run Shaw Hospital, Zhejiang University, Hangzhou, China

**Keywords:** radical mastectomy, intercostal lymph node, metastasis, case, surgical approach

## Abstract

The axillary lymph nodes are the primary group responsible for lymphatic drainage in the breast and, consequently, are the most common location for breast cancer metastasis. However, lymphatic pathways running from the breast, via intercostal spaces, to parasternal lymph vessels have also been identified. According to the American Joint Committee on Cancer eighth edition manual, regional lymph node metastasis normally travels to the ipsilateral axillary, supraclavicular, subclavicular, and internal mammary lymph nodes. The presence of intercostal metastasis is out the range of these regional lymph nodes. It is very rare for intercostal lymph nodes to be the extra-axillary site of metastasis in breast cancer, and it has been little reported on in the literature. Despite its rarity, it has the capacity to adversely affect the prognosis of breast cancer and drastically influence treatment choice. Here, we analyze such a case, with a patient receiving a radical mastectomy and metastatic intercostal lymph node dissection due to the presence of intercostal lymph node metastasis indicated via MRI. Furthermore, the potential application of preoperative 3-dimensional (3D) visualization and surgical planning is also discussed.

## Introduction

The predominant lymphatic drainage pathway drains from the breast toward the axilla (1). However, nodal metastases outside the axilla may be present in up to 56% of breast cancer patients (2). The sites of extra-axillary nodes include the internal mammary lymph nodes, the infraclavicular region, the supraclavicular fossa, the interpectoral (Rotter's) space, and the breast itself. Intercostal lymph node metastasis of breast cancer is an extremely rare extra-axillary site, a drainage pathway largely absent in literature. Although this drainage may be uncommon, it often escalates the severity of breast cancer and necessitates alternative treatment options.

Here, we reported on such a case. The case involves a patient receiving a radical mastectomy and metastatic intercostal lymph node dissection upon the discovery of intercostal lymph node metastasis indicated via MRI. Furthermore, the potential application of preoperative 3-dimensional (3D) visualization and surgical planning is also discussed.

## Case Report

A married, 51-year-old female felt the lump in the right breast 2 weeks prior to consultation. She had no significant medical history. Physical examination revealed a palpable mass at 6 o'clock in the right breast with a size of 2.0 × 1.5 cm, with no obvious lump in the left breast. The lump felt hard, with an indistinct border, no tenderness, and was movable. There was no skin involvement, orange peel, skin dimpling, or swollen lymph nodes under either armpit. Her body mass index (BMI) was 23.4 kg/m^2^.

**Figure 1:**
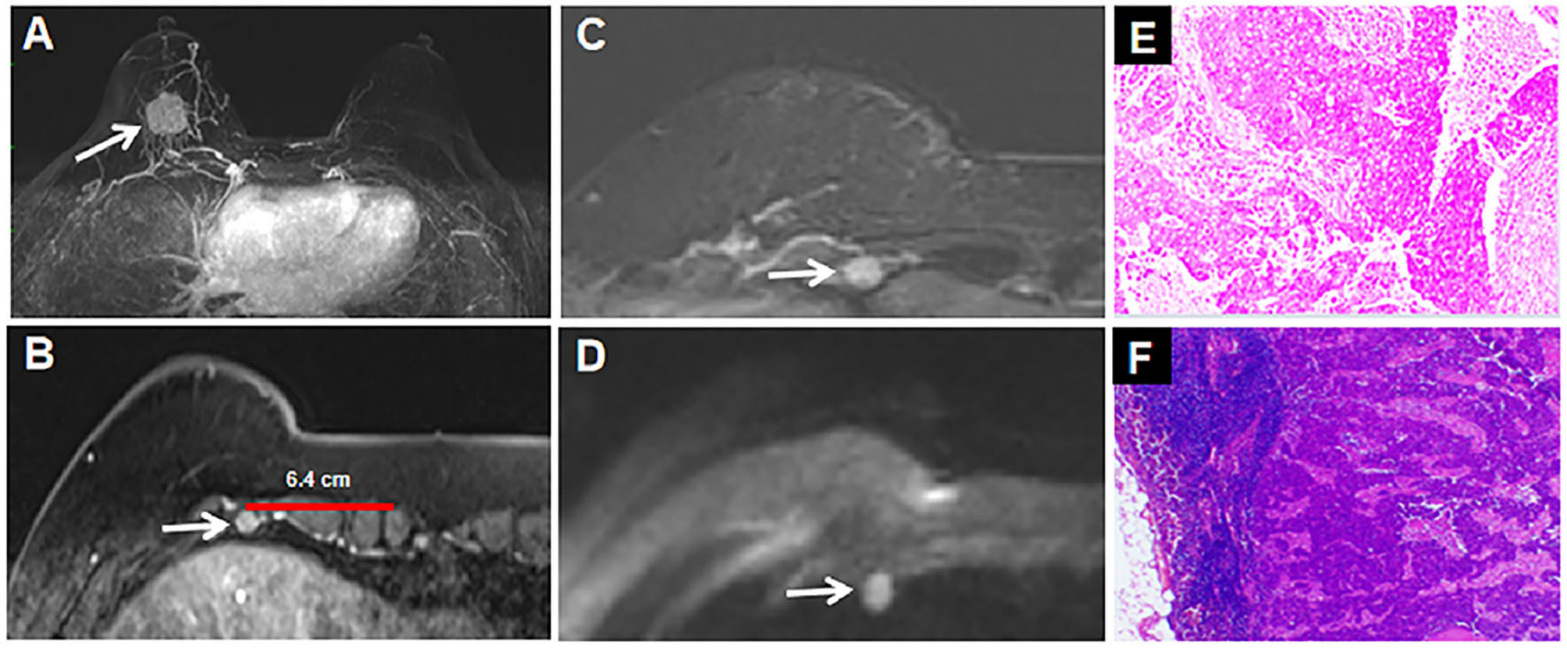
MRI of the breast tumor and intercostal lymph node. **(A)** MIP images show the irregular enhanced mass (2.4 × 2.3 × 2.4 cm) with focal and asymmetric vascularity at 6 o'clock in the right breast (white arrow). **(B)** T1-weighted contrast enhanced images show an oval 0.8 × 0.6 cm size, contrast-enhanced nodule in right fourth intercostal space (white arrow). **(C)** T2-weighted imaging indicate hyperintense of the nodule in right fourth intercostal rib (white arrow). **(D)** The nodule above is high signal on diffusion-weighted imaging (b-value=1,000 s/mm^2^, white arrow). **(E)** the breast mass was confirmed to be invasive breast cancer (non-special type, WHO III grade) by histopathology (Hematoxylin and eosin, original magnification, 100×). **(F)** The cancer cells were found in the right fourth intercostal nodule (Hematoxylin and eosin, original magnification, 100×).

An irregular hypoechoic mass, with a long diameter of 2 cm, was seen at the 6 o'clock position in the right breast on ultrasonography, which was highly suspicious for malignancy (BI-RADS category 4c). The preoperative MRI showed a spiculated mass in the right breast and an oval nodule in the right fourth intercostal region. A washout pattern on dynamic-enhanced sequence and restriction of diffusion on diffusion-weighted imaging were demonstrated both in the breast mass and the intercostal lymph node. The breast mass indicated malignancy (BI-RADS category 5), while the nodule in the right fourth intercostal rib was highly suspected to metastasize (Figures 1A–D).

**Figure 2:**
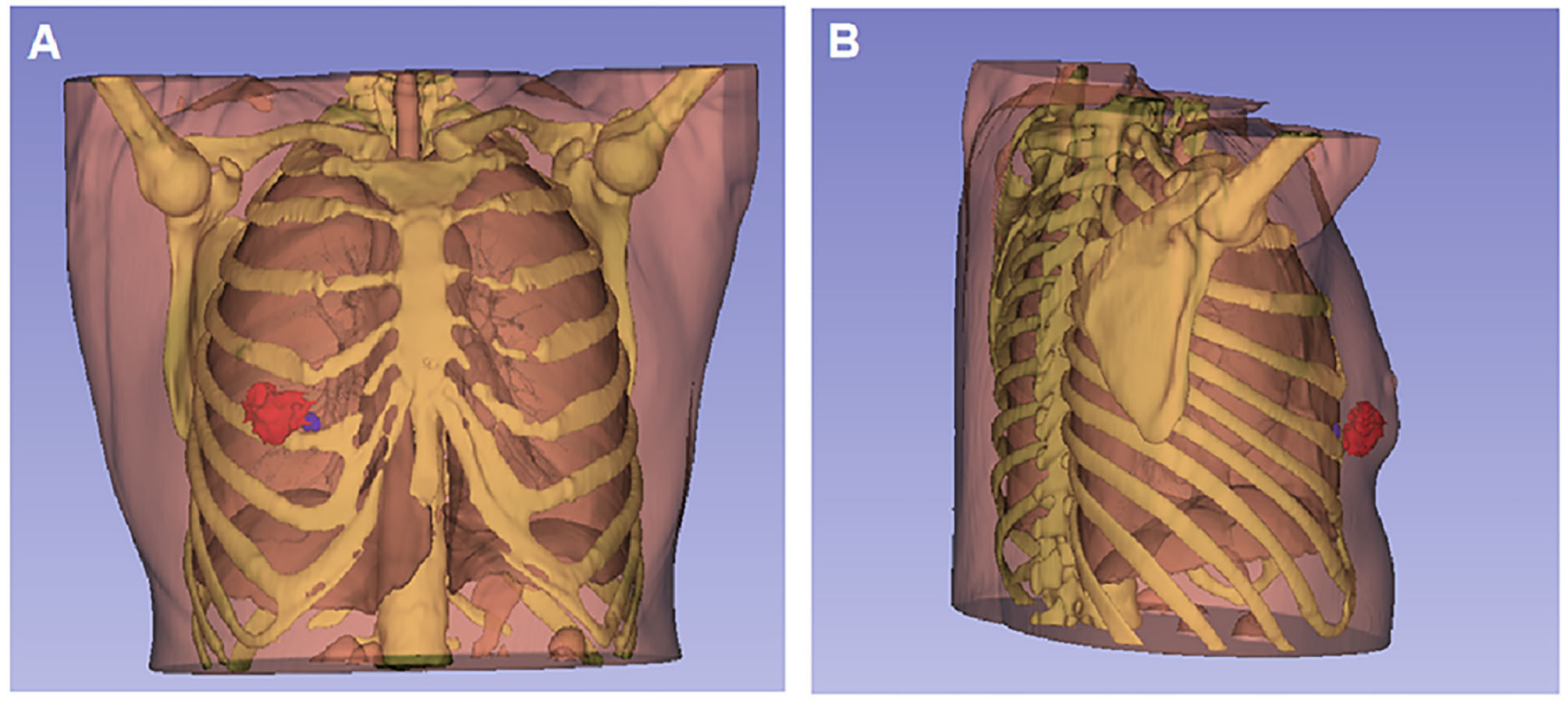
3D visualization and reconstruction of the breast tumor and intercostal lymph node. **(A)** The tumor mass (red mass) located at 5 and 6 o'clock positions in the right breast from the front view. **(B)** The metastatic intercostal lymph node (blue node) behind the tumor was close to the upper edge of the right fifth rib and adjacent to the right lower pleura from the right-side view.

3D visualization and reconstruction (https://download.slicer.org, version 4.10.2) showed the tumor mass (red mass) (Figure 2A), the intercostal lymph node (blue mass), and adjacent structure (Figure 2B).

She received a radical mastectomy, dissection of isolated metastatic intercostal lymph node and axillary lymph nodes, and postoperative adjuvant chemotherapy. The breast mass was confirmed to be invasive breast cancer by pathology (Figure 1E), while cancer cells were found in the right fourth intercostal nodule (Figure 1F) and two out of 12 resected axillary lymph nodes. On immunohistochemical analysis, the tumor cells were negative for estrogen and progesterone, and positive for human epidermal growth factor receptor 2 (HER2). The final stage of the patient was IIIA (pT1cN2bM0).

## Discussion

The axillary lymph nodes are the primary group responsible for breast lymphatic drainage and, consequently, are the most common location for metastasis (3). However, lymphatic pathways running from the breast via intercostal spaces to parasternal lymph vessels were found separate from typical metastatic sites (4). According to the American Joint Committee on Cancer eighth edition manual, regional lymph node metastasis includes the ipsilateral axillary lymph node, supraclavicular, subclavicular, and internal mammary lymph node; the presence of intercostal metastasis is out of the range of these regional lymph nodes (5). Intercostal lymph node metastasis may negatively affect the prognosis of the primary breast cancer patient, exacerbating breast cancer progression. Her final stage was IIIA (pT1cN2bM0), which was based on our clinical experience and the American Joint Committee on Cancer eighth edition manual (5). Unfortunately, accurate clinical staging requires large-scale assessment and long-term follow-up.

The intercostal lymph node metastasis found by preoperative imaging is a rare phenomenon. Intercostal lymph node metastasis of breast cancer has scarcely been reported in the literature and there is insufficient information on its effects. The best method for treating intercostal lymph node metastasis seems to be surgery or external radiation treatment. With external5 irradiation, it is difficult to give an adequate tumor dose to the intercostal lymph node without damaging surrounding tissues, especially in the lungs and the heart (6). The intercostal lymph node metastasis indicated by the preoperative images led to the final treatment decision.

3D reconstructions of this case were successfully performed, which generated 3D printed physical models. This 3D reconstruction and printing technology can aid in preoperative rehearsal, surgical planning, and the manufacturing of 3D implants, leading to improved surgical accuracy (7).

A radical mastectomy and metastatic intercostal lymph node dissection was performed as imaging revealed intercostal lymph node metastasis. The presence of intercostal lymph node metastasis is out of the range of the main groups of breast lymphatic drainage. Imaging evaluation of the lymph node metastasis of breast cancer should include intercostal lymph nodes in addition to the axillary, internal mammary, and supraclavicular lymph nodes. Limited research on this metastasis and danger to surrounding tissues complicates many traditional treatment approaches. The application of 3D visualization might be useful for preoperative evaluation, surgical planning, and 3D implants in the future.

## Data Availability Statement

The original contributions generated for the study are included in the article/Supplementary Material, further inquiries can be directed to the corresponding author/s.

## Ethics Statement

Written informed consent was obtained from the individual(s) for the publication of any potentially identifiable images or data included in this article.

## Author Contributions

YZ and JH took the lead in drafting the manuscript and provided magnetic resonance images. NM, LX, and JC provided supervision and participated in the literature review and in drafting the manuscript. All authors read and approved the final manuscript.

## Conflict of Interest

The authors declare that the research was conducted in the absence of any commercial or financial relationships that could be construed as a potential conflict of interest.
